# The role of microRNAs in neurobiology and pathophysiology of the hippocampus

**DOI:** 10.3389/fnmol.2023.1226413

**Published:** 2023-09-04

**Authors:** Seyed Khalil Rashidi, Ata Kalirad, Shahram Rafie, Ebrahim Behzad, Mitra Ansari Dezfouli

**Affiliations:** ^1^Department of Medical Biotechnology, Faculty of Medicine, Semnan University of Medical Sciences, Semnan, Iran; ^2^Department of Integrative Evolutionary Biology, Max Planck Institute for Biology Tübingen, Tübingen, Germany; ^3^Department of Neurology, School of Medicine, Ahvaz Jundishapur University of Medical Sciences, Ahvaz, Iran; ^4^Neuroscience Lab, Golestan Hospital, Ahvaz Jundishapur University of Medical Sciences, Ahvaz, Iran

**Keywords:** microRNA, hippocampus, neurogenesis, neural development, Alzheimer’s disease, epilepsy

## Abstract

MicroRNAs (miRNAs) are short non-coding and well-conserved RNAs that are linked to many aspects of development and disorders. MicroRNAs control the expression of genes related to different biological processes and play a prominent role in the harmonious expression of many genes. During neural development of the central nervous system, miRNAs are regulated in time and space. In the mature brain, the dynamic expression of miRNAs continues, highlighting their functional importance in neurons. The hippocampus, as one of the crucial brain structures, is a key component of major functional connections in brain. Gene expression abnormalities in the hippocampus lead to disturbance in neurogenesis, neural maturation and synaptic formation. These disturbances are at the root of several neurological disorders and behavioral deficits, including Alzheimer’s disease, epilepsy and schizophrenia. There is strong evidence that abnormalities in miRNAs are contributed in neurodegenerative mechanisms in the hippocampus through imbalanced activity of ion channels, neuronal excitability, synaptic plasticity and neuronal apoptosis. Some miRNAs affect oxidative stress, inflammation, neural differentiation, migration and neurogenesis in the hippocampus. Furthermore, major signaling cascades in neurodegeneration, such as NF-Kβ signaling, PI3/Akt signaling and Notch pathway, are closely modulated by miRNAs. These observations, suggest that microRNAs are significant regulators in the complicated network of gene regulation in the hippocampus. In the current review, we focus on the miRNA functional role in the progression of normal development and neurogenesis of the hippocampus. We also consider how miRNAs in the hippocampus are crucial for gene expression mechanisms in pathophysiological pathways.

## Introduction

MicroRNAs (miRNAs) are a group of non-coding RNAs that are typically about 22–25 nucleotides (nt). These small RNA molecules play a crucial role in regulating gene expression patterns (Coolen and Bally-Cuif, 2015). MicroRNAs are expressed in different organisms, such as humans, plants, fungi, bacteria and viruses. Lee and colleagues first identified these molecules in 1993 while investigating the non-coding transcript of *lin-4* in nematode *Caenorhabditis elegans* (Lee et al., 1993). Mature miRNAs are involved in the post-transcriptional regulation of gene expression through the repression of the translation of target mRNA or via degrading target mRNA. Functionally, miRNAs bind to their mRNA targets by relative complementarity between them and the 3’UTR of mRNA. Consequently, a single miRNA can suppress various mRNAs simultaneously, whereas a single mRNA may be suppressed by multiple miRNAs (Cho et al., 2019).

Studies show that miRNAs are important in regulating neurogenesis, neural differentiation, apoptosis and cell metabolism. These small RNAs are involved in the pathogenesis of many neurological and psychiatric diseases. They are important regulators involved in mechanisms of neurotoxicity, oxidative stress, inflammation, angiogenesis and damage to the blood–brain barrier. Thus, miRNAs have appeared as consequential mediators of the molecular pathways of various neurodegenerative disorders, including Alzheimer’s disease, Parkinson’s disease, stroke, epilepsy, amyotrophic lateral sclerosis, and Huntington’s disease. MicroRNAs can be present in physiological fluids including CSF, blood and sputum and can be studied as biomarkers for the diagnosis and treatment of neurological disorders (Khoo et al., 2012; Ricci et al., 2018; Wu et al., 2021).

The hippocampus is a complex structure located deep in the temporal lobe of the brain (Anand and Dhikav, 2012). The surgeon Julius Caesar Arantius first discovered this structure in 1587 (Anand and Dhikav, 2012). The hippocampus consists of two parts: Cornu ammonis known as hippocampus proper, and dentate gyrus (Anand and Dhikav, 2012). The hippocampus receive afferent connections from the amygdala, hypothalamus, prefrontal cortex, septum, and mammillary bodies and every stimulation of the nearby parts is reached to the hippocampus. Output signals from the hippocampus, pass through the fornix bundle to the anterior thalamus, hypothalamus, limbic system and cortex (Schultz and Engelhardt, 2014). The hippocampus is also very hyperexcitable tissue, meaning it can keep weak input signals into a long, sustained stimulation that helps to encode memory from different senses such as olfaction, visual, auditory, and tactile. The hippocampus is a core component of cognitive circuits involved in learning, memory and emotion. It is a plastic and vulnerable structure in the brain that is involved in multiple neurological and psychiatric disorders (Anand and Dhikav, 2012).

The hippocampus is a unique structure of the brain where neurogenesis occurs even in adulthood (Bonfanti and Peretto, 2011). The hippocampus has a central role in learning, memory, spatial navigation and emotional processing (Anand and Dhikav, 2012). The connections between the hippocampus and the neocortex are essential for purposeful behavior and conscious knowledge. There may be a complex balance between encoding memories in the hippocampus and retrieving reports from the frontal lobe (Morgado-Bernal, 2011). Alterations in hippocampal synaptic plasticity and hippocampal-dependent learning are important in functional connectivity changes in development and disorder (Duszkiewicz et al., 2019). The morphology of the developing neurons in the dentate gyrus that are originated from neural stem cells, is changed during differentiation. Their axons develop to form new functional synapses with mature neurons in the CA3 region. These connections integrated into the functional networks of hippocampal circuits. Also, neurogenesis in the dentate gyrus (DG) of the hippocampus is decisive in the formation and self-repair of central nervous system, as well as cognitive functions (Shenoy et al., 2015). These functions are influenced by the regulatory activity of miRNAs.

The normal hippocampal function depends on a series of adjusted complex processes that require exact spatial and temporal synchronization of gene expression at both the transcriptional and translational stages. The hippocampus is a miRNA-enriched part of the brain. Some miRNAs are specific to physiological processes in the developing neurons of the hippocampus, and many of them are dynamically expressed in the adult hippocampus, reflecting the need for different miRNAs in the hippocampus during life span (Cho et al., 2019). These miRNAs are decisive in the process of cell differentiation and cell fate through targeting the genes involved in intracellular signaling cascades (Smirnova et al., 2005). changes in the levels of the microRNAs can disrupt the regular pattern of gene expression and damage the stability and function of the hippocampal neurons (Cho et al., 2019). The pattern of miRNA expressions in the hippocampus is different in various cell types. For instance, miR-124 and miR-128 are mainly expressed in mature neurons of hippocampus but not in glial cells, while miR-23 is expressed in astrocyte cells (Smirnova et al., 2005), and miR-92b is particullary expressed in the neural stem cells (Tan et al., 2022). Astrocyte differentiation is influenced by Let-7b and miR-125 (Shenoy et al., 2015). The miR-17-92 and miR-124 are the regulator of neurogenesis and axonal growth pathways (Makeyev et al., 2007; Bian et al., 2013). miR-9 is the key element in controlling neural differentiation (Radhakrishnan and Anand, 2016), as well as axonal morphology and synaptic formation (Dajas-Bailador et al., 2012). Additionally, miR-219 (Dugas et al., 2010), miR-338 (Zhao et al., 2010), miR-138 (Dugas et al., 2010), miR-199a-5p (Letzen et al., 2010), and miR-145 [21]are important in the differentiation of oligodendrocytes. A decreased level of miR-125 caused apoptosis and an increased expression of miR-210 exacerbates neuronal damage (Wang et al., 2017). Post-injury inflammation is negatively regulated by miR-146a (Gaudet et al., 2018) that enhances oligodendrogenesis (Liu et al., 2017). Down regulation or suppression of miR-155 expression decreases post-injury inflammation (Roitbak, 2018). Following injury, Let-7b released from neurons and immune cells induces apoptosis, resulting in neuroinflammation (Mueller et al., 2014). Several miRNAs regulate various processes of cells in the nervous system. miR-25 inhibits the apoptosis of neurons and glia in the central nervous system (Peng et al., 2021). In addition, this microRNA increases cell proliferation and increases cell differentiation and migration and plays a role in brain development. miR-25 directly targets p57, which is one of the molecules involved in CDK inhibition, and stops the cell-cycle transition from S phase to G2 (Coolen and Bally-Cuif, 2015; Divisato et al., 2021).

The studies on the role of miRNAs in the hippocampus are still in their infancy. In the current review, we studied the role of miRNAs in the physiological function as well as pathophysiological pathways in the hippocampus.

## MicroRNAs are important regulators of neural development in the hippocampus

The hippocampus is an integral part of the complex neural networks of brain. Therefore, to ensure integration of functional connections, neural development in the hippocampus must be finely tuned. Subtle alterations in developmental processes in the hippocampus impair in its structure and function in a variety of pathways (Rolls, 2022). MicroRNAs (miRNAs) are key regulators of gene expression profile to determine the successive phases of neural development (Table 1; Coolen and Bally-Cuif, 2015). Conditional ablating of the miRNA maturation enzyme, Dicer, in the hippocampus during development results in reduced proliferation and increased apoptosis in neural progenitor cells, which severely disruption of hippocampal morphology (Li et al., 2011). Taken together, these abnormalities cause abnormal neural development and deficiency in brain connectivity and embryonic lethality (Kanellopoulou et al., 2005; Li et al., 2011; McLoughlin et al., 2012). Late embryonic deletion of Dicer reduces dendritic branch elaborations, increases the dendritic spine length and induces neurodegeneration (Davis et al., 2008).

**Table 1:** Hippocampal microRNAs (miRNAs) in neurogenesis and neural development.

miRNA	Target	Pathway	Ref
miR-132	MECP2	Dendritic growth and arborization	(Magill et al., 2010; Mendoza-Viveros et al., 2017)
miR-124a	NeuroD1, Notch,LHX2	Apoptosis, axonal development	(Sanuki et al., 2011; Konrad and Song, 2021)
miR-134	LIMK1	Synaptic compartments, dendritic spine formation	(Katsu et al., 2019)
miR379-410	MEF2	Dendritic outgrowth, synaptic formation	(Fiore et al., 2009)
miR-210	SNAP25	Dendritic arbor spine density	(Ren et al., 2018; Watts et al., 2021)
miR-9	REST, NR2E1, SIRT1, FOXG1	Dendritic growth, synaptic transmission, neurogenesis	(Giusti et al., 2014)
MiR-19	RAPGEF2	Cell migration	(Han et al., 2016)
miR-200	ZEB1 and ZEB2, PRKAR2B	Cell proliferation, synaptic formation	(Beclin et al., 2016; Weise et al., 2019)
miR-153	JAGGED1 and HEY2	Neurogenesis, glycogenesis	(Qiao et al., 2020)
miR-184	NUMBL	Proliferation and differentiation	(Liu et al., 2010; Lang and Shi, 2012)
miR-125	BMF,LIN28	Neurogenesis, neuronal survival	(Cui et al., 2012)
miR-29a	FSTL1	Neuronal morphology and neurite outgrowth	(Ma et al., 2020)

MECP2, methyl-CpG binding protein 2; LHX2, LIM homeobox 2; LIMK1, LIM domain kinase 1; MEF2, myocyte enhancing factor 2; SNAP25, synaptosome associated protein 25; FOXG1, forkhead box protein G1; REST, RE1 silencing transcription factor; NR2E, nuclear receptor subfamily 2 group E member 1; SIRT1, silent mating type information regulation 2 homolog 1; RAPGEF2, Rap guanine nucleotide exchange factor 2, ZEB, zinc finger E-box binding homeobox; PRKAR2B, protein kinase cAMP-dependent type II-Beta regulatory subunit; HEY2, Hairy/enhancer-of-split related with YRPW motif protein 2; NUMBL, NUMB like endocytic adaptor protein; BMF, Bcl2 modifying factor; FSTL1, Follistatin like 1.

MicroRNA-132 is essential for dendritic growth and arborization of developing neurons in the hippocampus through its regulatory effect on CREB signaling pathway (Magill et al., 2010). MiR-124a is required for apoptosis prevention and the appropriate axonal morphology of the hippocampal neurons (Sanuki et al., 2011). Interestingly, miR-134 is involved in determining the morphology of synaptodendritic junctions of excitatory synapses in hippocampal neurons. This activity of miR-134 is the result of repression of the mRNA coding for the LIMK1 protein kinase, which regulates the spine formation (Schratt et al., 2006). Extracellular stimuli, such as brain-derived neurotrophic factor(BDNF), alleviate the miR-134 suppression on LIMK1 expression, which triggers synaptic development, maturation and plasticity (Schratt et al., 2006). The activities of hippocampal neurons increase the expression of a large cluster of brain-specific miRNAs called miR379-410. In fact, increasing neuronal activity induces the binding of the transcription factor myocyte enhancer factor 2 (Mef2) to the promoter region of this cluster to increase transcription. The expression of miRNAs of the miR379-410 cluster is essential for the dendritic growth and the formation of synapses in the hippocampal neurons (Fiore et al., 2009). It has been reported that miR-329-3p and miR-495-3p induce homeostatic synaptic depression (HSD) and inhibit chronic increases in network activity. These miRNAs inhibit proline-rich protein 7/transmembrane adapter protein 3 (Prr7) which is the protein involved in the stability of excitatory synapses and the size of dendritic spines at the synaptic site through protection of synaptic proteins (Inouye et al., 2022).

It has been reported that miR-125 is involved in neural differentiation through down regulation of LIN28. LIN28 is codding an important post-transcriptional regulator protein that has a critical role in growth timing and self-renewal. This protein prevents the final maturation of LET7 microRNAs family, which are the regulators in cell development and differentiation (Galagali and Kim, 2020). In addition, miR-125 inhibits apoptosis by targeting BMF expression. This gene encodes a protein of Bcl2 family members and is involved in recognition of intracellular damage and initiating apoptosis (Tamtaji et al., 2022).

Watts et al. reported that miR-210 is localized in somas and dendritic processes of the hippocampal neuron. Moreover, the expression level of miR-210 increases following neural stimulation. In hippocampal neurons, loss of miR-210 increased oxidative phosphorylation and ROS generation in hypoxia situation and increases dendritic arbor spines in the hippocampus (Watts et al., 2021). Moreover, miR-210 knockout in mice displayed deficits in behavioral and learning abilities. These observations suggested a conserved and stimuli-dependent role for miR-210 in neural development and cognition (Watts et al., 2019, 2021). It has been reported that miR-17-92 cluster induces neurogenesis as well as neuronal differentiation in the dentate gyrus (DG) through down regulation of a cytoskeleton associated protein, Enigma homolog 1 (Enh1) (Pan et al., 2019).

In addition to controlling the morphology of neurons and synaptic sites, microRNAs can also regulate neural migration in the hippocampus. Neuronal migration is key to the brain development. In this process, cells move from their place of origin to their site of integration (Faini et al., 2021). Although it seems that extensive neuronal migration is specific to embryonic stages and the beginning of birth, neuroblasts generation and migration occurs throughout life in the dentate gyrus of the hippocampus (Bressan and Saghatelyan, 2021). Several miRNAs are prominent in the migration of neurons. It has been reported that miR-9 promotes neurogenesis but inhibits migration of neural progenitors through targeting and repressing stathmin, a protein that stimulates microtubule degradation (Delaloy et al., 2010). Moreover, miR-9 expression is critical for convenient dendritic growth and synaptic transmission through targeting of REST which encodes a transcriptional repressor protein (Giusti et al., 2014). The expression levels of miR-19 increase in neural stem cells and decrease during the development and maturation of hippocampal neurons. This microRNA controls neuronal migration process by suppressing RAPGEF2 (Han et al., 2016). Aberrant expression of miR-19 in the hippocampus causes dysregulated migration of developing neurons (Han et al., 2016). In addition, miR-19 plays a key role in the positioning of the hippocampal tissue via Wnt-mediated signaling pathway (Sindhu et al., 2019). It has been demonstrated that miR-29a, in addition to regulate neural morphology and soma size, targets mRNAs related to extracellular matrix proteins including Fibrillin1, Follistatin-like1(FSTL1) and Laminin subunit gamma-2 to control neuronal migration (Ma et al., 2020). Additionally miR-29 modulates chromatin methylation mediated by Dnmt3a, an essential process in normal brain maturation (Swahari et al., 2021). Other microRNAs including miR-125b, miR-129, miR-934, miR-26a, mir-137 and let-7d are also involved in migration of the newborn neurons (Cui et al., 2012; Zhao et al., 2013; Mahmoudi and Cairns, 2017; Wu et al., 2019; Guo et al., 2020; Prodromidou et al., 2020; Sun et al., 2020).

## MicroRNAs and neurogenesis in the hippocampus

Neurons, astrocytes and oligodendrocytes in the central nervous system are generated from neural stem cells (NSCs) which are self-renewing, multipotent progenitor cells (Stappert et al., 2018). During developmental stages, the entire nervous system is originated from the proliferation and migration of NSCs. However, in the adult mammalian brain, the two main neurogenic niches, namely the subventricular zone (SVZ) and the subgranular zone (SGZ) of the dentate gyrus of the hippocampus, continue to contain NSCs (Obernier and Alvarez-Buylla, 2019). Neurogenesis in the hippocampus is critical in learning and memory, aging, and neurodegenerative processes. The proliferation of NSCs is controlled by both internal genetic and epigenetic elements and external environmental cues transduced by the NSC niche (Zhao and Moore, 2018). Aberrant neurogenesis in the hippocampus may lead to neurological disorders including Alzheimer and epilepsy. Consequently, extensive studies have been conducted in the past decades to understand the regulation intricacies of neurogenesis in the hippocampus (Stappert et al., 2018; Zhao and Moore, 2018; Pan et al., 2019).

Many physiological factors including physical activities, environmental situations, cognitive functions and aging can affect neurogenesis in the hippocampus (Stappert et al., 2018). Environmental enrichment activates the cell cycle, induces neurogenesis, and increases neuronal survival in the hippocampus (Barak et al., 2013; Zhao and Moore, 2018; Lee et al., 2023). Evidently, miRNA expression profile in the hippocampus undergoes relatively large changes following exposure to an enriched environment (Barak et al., 2013). In total, 29% of miRNAs in the hippocampus were down-regulated while 8% were up-regulated after exposure to the enriched environment (Barak et al., 2013; Stappert et al., 2018). miR-132 exhibits the most drastic up regulation in response to environmental enrichment among the hippocampal miRNAs (Barak et al., 2013). Interestingly, miR-132 is found to be an essential mediator in neural precursors proliferation in the adult hippocampus (Hansen et al., 2016; Walgrave et al., 2021). In addition, miR-132 is downregulated in Alzheimer’s disease(AD) pathobiology and replenishing this microRNA in adult mouse with AD restores the hippocampal neurogenesis and alleviates the memory impairment (Walgrave et al., 2021). These findings corroborate the importance of hippocampal neurogenesis and hint at the therapeutic potential of targeting miR-132 as a therapeutic strategy for neurodegeneration (Walgrave et al., 2021).

A crucial aspect of the regulation of NSCs proliferation, from embryo to adult, is regulated by microRNAs (Lang and Shi, 2012). It has been demonstrated that miR-9 transcript level varies from the embryonic stage during maturation and to aging (Smirnova et al., 2005; Delaloy et al., 2010). Study on adult neurogenesis illustrated the enrichment of miR-9 in the quiescent NSCs in the brain. This microRNA in the miR-9-Ago complex is transported to the nucleus by TNRC6 shuttle protein. The presence of miR-9 in the nucleus is essential for NSC quiescence through the enhancement of Notch signaling (Katz et al., 2016). The concentration of miR-9 in the nucleus seemingly increases during aging. Consequently, miR-9 concentration in the nucleus was detected in the NSCs in the neurogenic niches in the mouse brain as well, showing a conserved role for miR-9 in regulation of NSC quiescence. During neural differentiation, the expression level of miR-9 and the expression level of nuclear receptor TLX (or NR2E1) change inversely (Zhao et al., 2009). TLX protein is essential for preservation of NSCs in undifferentiated conditions for cell proliferation (Kandel et al., 2022). The TLX expression is suppressed by miR-9 via base pairing to the 3′ UTR of TLX mRNA. Moreover, TLX represses the expression of the miR-9 pri-miRNA (Zhao et al., 2009). The up-regulation of miR-9 significantly reduces the NSC proliferation and promotes differentiation process in neuron and glia. Interestingly, miR-9 knockdown promotes proliferation of NSCs. Intrauterine electroporation of miR-9 leads to precise neuronal differentiation and neuronal migration (Zhao et al., 2009). Interestingly, In mouse embryonic neural stem cells, miR-9 inhibits neurogenesis and induces differentiation by targeting Sirtuin 1 (SIRT1) (Saunders et al., 2010). Moreover, miR-9and suppresses neurogenesis and promotes neural differentiation by targeting Forkhead box G1 (FOXG1) (Shibata et al., 2011). Foxg1 is a transcription factor that induces lineage progression and is involved in hippocampal neurogenesis (Wang et al., 2022). It has been demonstrated that Foxg1 affect the biogenesis of miR-200 via interaction with Ddx5. Ddx5 protein is involved in RNA maturation with ATP-dependent RNA helicase activity (Xing et al., 2019). In the hippocampus, both Foxg1 and Ddx5 link to the microprocessor complex, to recruit DROSHA for miR-200 maturation (Weise et al., 2019). Finally, miR-200 family members regulates neurogenesis and synaptic maturation via targeting key regulators of cell cycle. Zeb1 and Zeb2 are transcription factors involved in cell proliferation and are suppressed by miR-200 (Beclin et al., 2016). Moreover, miR-200 targets cAMP-dependent protein kinase type II-beta regulatory subunit (PRKAR2B) in the hippocampal neurons which is involved in synaptic formation (Weise et al., 2019).

Qiao and collaborators evaluated the expression levels of miR-153 various mouse tissues and they reported high expression of this microRNA expression in the nervous system, particularly in the hippocampus (Qiao et al., 2020). This miRNA is a highly conserved microRNA in mammals and is involved in adult neurogenesis (Lang and Shi, 2012). Moreover, miR-153 expression level is reduced in the in the hippocampus during aging and leads to declined hippocampal neurogenesis and behavioral impairment. However, miR-153 overexpression in the hippocampus of aged mice increased neurogenesis and markedly improved the cognitive functions (Qiao et al., 2020). Studies demonstrated that miR-153 induced neurogenesis via inhibitory effect on Notch signaling pathway. The Notch pathway plays critical roles in the preservation, proliferation, and differentiation of NSCs in the hippocampus (Gómez-Pinedo et al., 2019). However, miR-153 critically inhibits glycogenesis in the hippocampus (Qiao et al., 2020). This microRNA increases the expression level of neuron-specific γ-enolase (NSE), neuronal nuclei (NeuN), and N-ethylmaleimide-sensitive fusion attachment protein 23 (SNAP23) and SNAP25 in hippocampal neurons. Interestingly, miR-153 significantly up regulates the expression of Peroxiredoxin 5 (PRX5), which encodes a protein that preserved neurons from apoptosis processes. Based on these findings, miR-153 in the hippocampus may prove a prospective target against neurodegenerative mechanisms (Xu et al., 2019). Ethanol decreases miR-153 expression in NSCs in line with disruption of neurogenesis and differentiation pattern hinting at the role of this microRNA as a mediator in ethanol-induced teratogenesis (Tsai et al., 2014).

MiR-184 enhances neurogenesis and inhibits neural differentiation (Lang and Shi, 2012). Methyl CpG binding protein 1 (Mbd1) modulates the expression of miR-184 in NSCs (Liu et al., 2010). Interestingly, miR-184 level increases in MBD1-deficient NSCs. It has been shown that Mbd1 binds to the miR-184 loci in the methylated regions and inhibit its transcription by the formation of silent chromatin. miR-184 down regulates the expression of Numb like (NUMBLl) gene which encodes a protein involved in regulation of neural development (Petersen et al., 2002; Liu et al., 2010). The overexpression of NUMBLl compensates for the effect of the miR-184 overexpression or MBD1 knockout, leads to neurogenesis inhibition and induces in neural differentiation in the hippocampus. This result indicated that miR-184 induces neurogenesis through targeting NUMBL1 expression in hippocampal NSCs (Liu et al., 2010; Lang and Shi, 2012). The frequently studied microRNAs in the neurogenesis and neurodevelopment of the hippocampus are listed in Table 1.

## Hippocampal microRNAs in AD pathogenesis

Alzheimer’s disease (AD) is currently recognized as the most prevalent cause of dementia in the elderly population. Memory problems and cognitive decline are the main complications of AD. Consequently, the AD patients are unable to perform daily functions independently (Kou et al., 2020). AD is characterized by three significant abnormalities in the hippocampus and neocortex areas of the brain. Firstly, the brain in these areas atrophies due to loss of neurons, as open gyri and large ventricles are commonly seen in the brain morphology a prognosis that turns AD into a neurodegenerative disease (DeTure and Dickson, 2019). Secondly, post-mortem brains reveals extracellular plaques of dense protein aggregation called amyloid beta in the hippocampus and neocortex areas. Around extracellular amyloid deposits, axons and dendrites are deformed and synaptic terminals are disrupted. Amyloid beta deposition is associated with inflammatory responses of astrocytes and microglia (Kou et al., 2020). Amyloid plaques also have been observed in the walls of vessels in the brain of the AD patients[83; 84]. Thirdly, neurons have abnormalities in the structure of the cytoskeleton, the most significant of which are neurofibrillary tangles. These tangles are filamentous inclusions of hyper-phosphorylated isoforms of tau protein, a microtubule associated protein (MAP) that is normally soluble.

Aβ is generatated by the proteolysis of amyloid precursor protein (APP) that is a large transmembrane glycoprotein (DeTure and Dickson, 2019). When APP is cleaved by β-secretase and γ-secretase, Aβ is produced (amyloidogeneic pathway). In the non-amyloid genetic pathway, α-secretase cleaves the APP protein within the Aβ amino acid sequence. This cleavage prevents the formation of Aβ peptides (Kandel et al., 2000).

Aβ production level is closely related to the expression levels of APP and BACE1 (Coulson et al., 2010; DeTure and Dickson, 2019; Kou et al., 2020). miR-101, miR-20a, and miR-17 inhibit APP expression and down regulation of these miRNAs has important therapeutic effect on the progression of AD (Zoltowska et al., 2020; Samadian et al., 2021; Liu et al., 2022). Decreased levels of microRNAs miR-29a/b-1, miR-195, miR-339-5p, miR-15b, miR-188-5p, miR-124, miR-22-3p and miR-374b-5p has been reported in the hippocampus of AD patients (Kou et al., 2020; Xia et al., 2022). On the contrary, BACE1 mRNA expression is enhanced in the hippocampus of AD patient (Coulson et al., 2010). In the subsequent experiments, BACE1 mRNA expression was reduced and Aβ aggregation was decreased following the transfection of these microRNAs in Aβ-induced AD cell models (Kou et al., 2020; Zoltowska et al., 2020). Moreover, miR-149, miR-34a-5p, miR-16, miR-29c and miR-124 have been unequivocally shown to target BACE1. The expression level of BACE1 mRNA was reduced and Aβ accumulation was decreased after the transfection of these miRNAs in the AD affected hippocampal neurons (An et al., 2017; Liu et al., 2022). In a study on the SAMP8 mouse model of Alzheimer’s diseases, the expression level of miR-340 was decreased, while BACE1 was increased, indicating the opposite correlation between the levels of miR-340 and BACE1 in the hippocampus of AD mice (Liu et al., 2022). Crucially, the overexpression of miR-340 inhibites the expression of BACE1. The binding potential between miR-340 and BACE1 was confirmed using dual-luciferase reporter assay (Tan et al., 2020). miR- 340 reduces the aggregation of amyloid-β and inhibited neuronal apoptosis via targeting BACE1 (Tan et al., 2020). Moreover, miR-31 regulatory effect on APP and BACE1 provides a potential mechanism against AD. Interestingly, miR-31 expression significantly decreased in AD patients. The overexpression of this miRNA ameliorated AD neuropathology in the AD transgenic mice, and consequently, reduced Aβ accumulation in the hippocampal and subiculum structures. The increasing levels of miR-31 leads to a significant alleviation of memory deficits and cognitive dysfunctions (Barros-Viegas et al., 2020; Li et al., 2023).

**Figure 1:**
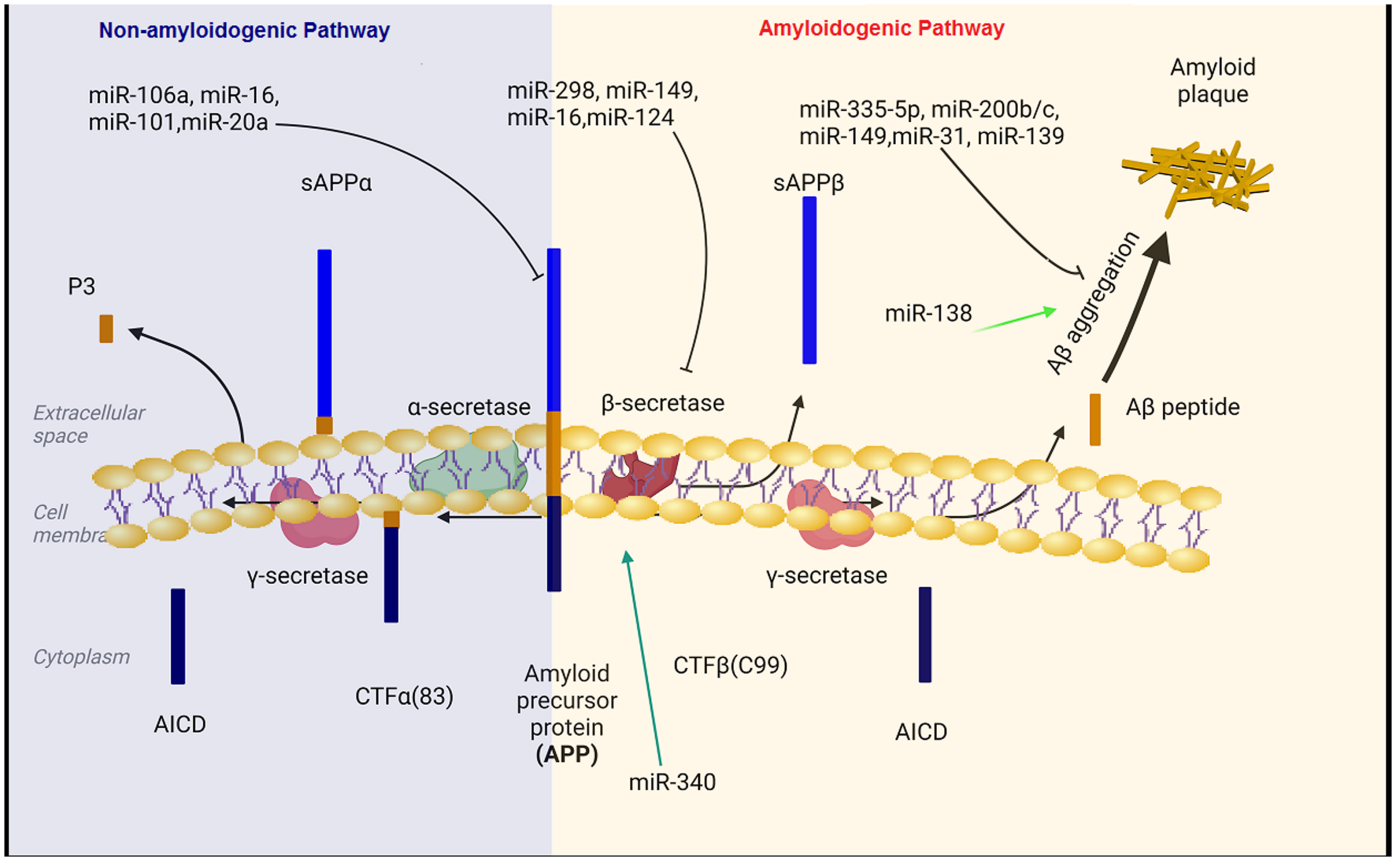
The regulatory role of miRNAs in the production and accumulation of amyloid-beta. Aβ pathogenic peptides are produced by proteolytic cleavage of APP by β-secretase (BACE1) and γ-secretase complex. Aβ peptides formed through the amyloidogenic pathway can aberrantly accumulate in plaques, which are important hallmarks in the pathology of Alzheimer’s disease. MicroRNAs can affect the formation and aggregation of Aβ at different levels. BACE1, beta-site amyloid precursor protein cleaving enzyme; APP, amyloid precursor protein.

Hegaki et al. found that amyloid beta (Aβ) production in AD neurons up regulates miR-200b/c. In the hippocampus miR-200b/c decreased secretion of the Aβ peptide. Additionally, overexpression of miR-200b/c in the brain improved cognitive problems that caused by intracerebroventricular accumulation of the oligomeric Aβ. This miRNA targets ribosomal protein S6 kinase B1 (S6K1). S6K1 is a negative regulator of insulin receptor substrate 1. Taken together, miR-200b/c alleviates insulin resistance via targeting S6K1 and inhibits Aβ secretion as well as Aβ-induced neurotoxicity by activating the insulin signaling (Higaki et al., 2018). In the brain tissue of AD patients, miR-132/212 and miR-335-5p was dramatically decreased. Down regulation of miR-132/212 increased the generation of the Aβ peptide and the accumulation of plaques. Moreover, miR-132/212 reduces the Aβ secretion via targeting SIRT1 (Hernandez-Rapp et al., 2016). In APP/PS1 mice and SH-SY5Y/APP cells AD symptoms were significantly improved after the up regulation of miR-335-5p, since miR-335-5p suppresses the JNK3 signaling cascade, thus reducing Aβ generation (Kou et al., 2020). Furthermore, miR-335-5p levels were decreased in the brain tissue of patients affected with AD whereas the expression of miR-335-5p and JNK3 were reversely correlated in these patients (Wang et al., 2020). Overexpression of miR-138 in the brain impairs cognitive functions and increases anxiety in mice. MiR-138 up regulation *in vivo* enhanced Aβ production and altered synaptic functions and induced inflammation in the hippocampus via targeting the SIRT1 expression (Boscher et al., 2019; Liu et al., 2022). Additionally, miR-26a-5preduced DYRK1A expression and inhibited Aβ aggregation (Figure 1; Liu et al., 2022).

**Figure 2:**
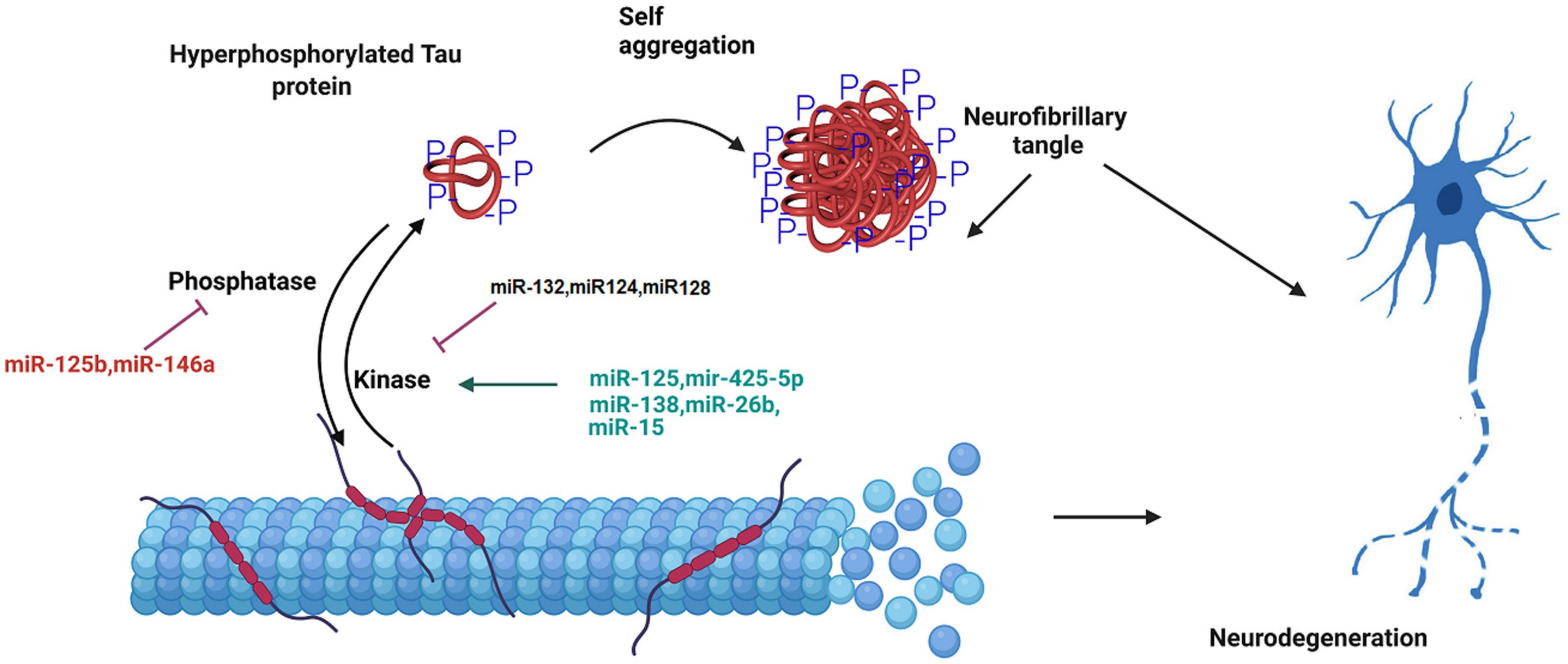
The role of miRNA in the formation of neurofibrillary tangles. Tau is a microtubule-associated protein that undergoes hyperphosphorylation in AD pathology. The phosphorylated tau protein is released from the microtubule. This disrupts the structure and integrity of microtubule in the neuron. The aggregation of hyperphosphorylated tau proteins causes the formation of neurofibrillary tangle. MicroRNAs regulate tau hyperphosphorylation and neurofiblirary tangle formation, and contribute to the progression of Alzheimer’s disease.

Another important hallmark of AD is hyperphosphorylation of tau protein. In a research on miRNA expression analysis in the brain tissues of AD patients compared to the healthy group, miR-125b was found to dramatically increased, and the levels of miR-124 and miR-132 was decreased. miR-125b induces the tau protein phosphorylation and neuronal apoptosis by targeting Forkhead Box Q1 (Liu and Zhang, 2019; IMa et al., 2022). The expression of miR-132 correlated with tau accumulation and cognitive problems in AD. Deficiency in miR-132 increases the expression, the phosphorylation and the accumulation of tau protein. Functionally, miR-132 regulates tau expression by directly targeting its mRNA. Moreover, miR-132 improved cognitive deficits and tau metabolism in AD mice (Smith et al., 2015; El Fatimy et al., 2018). In addition, miR-132 reduces total, phosphorylated, acetylated, and cleaved forms of tau protein involved in tauopathy, improves neurite morphology, and lowers neuronal apoptosis (El Fatimy et al., 2018). Additionally, miR-132 preserves neurons against the amyloid β-peptide (Aβ) accumulation and glutamate stimulation. miR-132 regulates the tau modifiers, acetyltransferase EP300 and kinase GSK3β (El Fatimy et al., 2018). Furthermore, miR-124-3p attenuates tau protein hyperphosphorylation by targeting Caveolin-1 expression and supressing Caveolin-1-PI3K/Akt/GSK3β pathway in AD (Kang et al., 2017). The overexpression of miR-425-5p enhances tau phosphorylation, activates glycogen synthase kinase-3β (GSK-3β), and induces apoptosis via targeting Heat shock protein B8 (HSPB8). This miRNA was up regulated in AD patients and HEK293/tau mutant cells (Yuan et al., 2020). Moreover, microRNA-128 suppressed tau phosphorylation and reduced amyloid beta aggregation via targeting GSK3β, APPBP2, and mTOR in Alzheimer’s disease. Up regulation of miR-128 in the hippocampus of AD mice ameliorated learning and memory deficits, decreased plaque aggregation, and increased autophagy flux (Figure 2; Yuan et al., 2020).

Various researches have demonstrated that several single-nucleotide polymorphisms (SNPs) in miRNAs correlate with the risk AD. Bucher et al. screened 546 cases of early-onset AD by whole exom sequencing. They identified 86 copy number variants in miRNA-encoding genes, of which miR-138-2 duplication were present only in early-onset AD cases. Because of the role of miR-138 in the Aβ accumulation and tau phosphorylation, this miRNA is involved in the risk of early-onset AD (Boscher et al., 2019). A research focused on evaluation of the genotype and alleles of miR146a polymorphism in a population consisting of 292 AD patients and 300 healthy controls. In this study, a difference in allelic frequencies of rs57095329 was seen between AD cases and controls, where the AA genotype of rs57095329 increased the incidence of AD. In cell culture, the AA genotype of rs57095329 exhibited increased expression of IL-6 and IL-1β in response to inflammation (Cui et al., 2014). Notably, Zhang et al. found that polymorphism in miR-146a, rs2910164 is correlated with the genetic predisposition to AD. The rare C allele of this polymorphism is more frequent in AD patients and is involved in upregulation of tumor necrosis factor-α (TNF-α) following β-amyloid toxicity (Zhang et al., 2015). Finally, Ghanbari et al. identified rs2291418 in the miR-1229 precursor to be significantly associated with AD using genome wild association analysis. This variant enhances the production of miR-1229-3p (Ghanbari et al., 2016) and change the expression level of SORL1, a gene that is involved in AD. In addition, among 42,855 polymorphisms in miRNA-binding sites, they found 10 polymorphisms which are associated with susceptibility to Alzheimer’s disease, including rs6857, which increases the miR-320e- effect on PVRL2 expression (Ghanbari et al., 2016). Table 2 showed important hippocampal miRNAs involved in AD pathology.

**Table 2:** Hippocampal microRNAs (miRNAs) in AD.

miRNA	Level in the hippocampus	Target	Pathway to AD	Ref
miR-101, miR-20a	Decreased	APP	Amyloid-β production	Fan et al. (2010), Vilardo et al. (2010)
miR-149, miR-34a, miR-16, miR-29c	Decreased	BACE1	Amyloid-β production	Kou et al. (2020), Samadian et al. (2021)
miR-340	Decreased	BACE1	Amyloid-β accumulation, cell apoptosis	Tan et al. (2020)
miR-31	Decreased	APP, BACE1	Aβ production	Barros-Viegas et al. (2020)
miR-200b/c	Decreased	S6K1	Insulin signaling, amyloid-β secretion	Higaki et al. (2018)
miR-132/212	Decreased	SIRT1	Aβ production and senile plaque deposition	Hernandez-Rapp et al. (2016)
miR-335-5p	Decreased	JNK3	Amyloid-β accumulation	Wang et al. (2020)
miR-138	Increased	SIRT1	Aβ production, synaptic functions, inflammation	Boscher et al. (2019), Lu et al. (2019)
miR-26a-5p	Decreased	DYRK1A	Tau phosphorylation and Aβ accumulation	Liu et al. (2020)
miR-125b	Increased	Bcl-W, DUSP6, PPP1CA, FOXQ1	Tau hyperphosphorylation, neuronal apoptosis, inflammation	Banzhaf-Strathmann et al. (2014), Zhuang et al. (2020)
miR-132	Decreased	RBFOX1, GSK3β, EP300, and Calpain 2	Tau phosphorylation, neurite elongation, glutamate excitotoxicity	Liu et al. (2022), El Fatimy et al. (2018)
miR-128	Decreased	GSK3β, APPBP2, and mTOR	Tau phosphorylation, amyloid-β accumulation	Li et al. (2023)
miR-425-5p	Increased	HSPB8	Tau hyperphosphorylation, glycogen synthase kinase-3β (GSK-3β) activity, and apoptosis	Yuan et al. (2020)
miR-124-3p	Decreased	Caveolin-1	Tau hyperphosphorylation, apoptosis	Kang et al. (2017)

APP, amyloid beta precursor protein; BACE1, Beta-secretase 1; S6K1, ribosomal protein S6 kinase Beta-1; SIRT1, silent mating type information regulation 2 homolog 1; DYRK1A, dual-specificity tyrosine phosphorylation-regulated kinase; JNK3, c-jun-N-terminal kinase 3; CXC10, C-X-C motif chemokine ligand 10; Bcl, B-cell lymphoma; DUSP6, dual specificity phosphatase 6; PPP1CA, protein phosphatase 1 catalytic subunit Alpha; RBFOX1, RNA binding Fox-1 homolog 1; GSK3β, glycogen synthase kinase 3 Beta; APPBP2, amyloid beta precursor protein binding protein 2; mTOR, mammalian target of rapamycin; HSPB8, heat shock protein B8.

## Hippocampal miRNAs in epilepsy

Epilepsy is caused by the series of transient abnormality, resulting from aberrant, highly synchronous firing in the brain functional networks. Recurrent convulsions and decreased level of consciousness are common clinical manifestation of epilepsy, symptoms that dramatically influence the lifestyle, daily functions, and physical and psychological condition of the patients (Wang and Zhao, 2021). Alteration in the gene expression pattern result in changes in neuronal firing threshold in epilepsy. Processes related to apoptosis, neurodegeneration and inflammation and oxidative stress, which are affected by changes in gene expression, are crucial for development of diseases in the brain (Dichter, 2009; Ghafouri-Fard et al., 2022). This abnormal brain function is mediated by changes in transcription factors, epigenetic pathways such as DNA methylation and histone modifications and post-transcriptional regulation. The signaling cascades involved in excitotoxicity and the underlying causes of abnormal apoptotic pathways in neurons, aberrant regeneration of glial cells, and inflammatory immune responses form complex networks of interactions. miRNAs play a role as important regulators of apoptotic processes, regenerative pathways and immune responses in the pathobiology of epilepsy (Wang and Zhao, 2021; Ghafouri-Fard et al., 2022).

Studies on animal model of epilepsy and human tissues demonstrate morphological changes, including loss of dendrites and axons in the hippocampus. In the pathology of temporal lobe epilepsy, irregular expression of genes in the hippocampus is involved, and miRNAs play a significant role in modulating gene expression (Table 3; Dichter, 2009). Lourenço Antônio and colleague have shown that the expressions of miR-145, miR-181c, miR-199a and miR-1183 differed quantitatively in the hippocampus and circulation of patients with mesial temporal lobe epilepsy with hippocampal sclerosis (MTLE-HS) in comparison to the healthy individuals. This alteration was most prominent for miR-145, which was decreased in the hippocampus and increased in the blood of MTLE-HS cases. They considered these microRNAs as diagnosis and prognosis biomarkers of epilepsy (Antônio et al., 2019).

**Table 3:** Hippocampal microRNAs (miRNAs) in epilepsy.

miRNA	Level in the hippocampus	Target	Pathway to epilepsy	Ref
miR-187	Decreased	IL10, SPRY1	Inflammation, apoptosis	Rossato et al. (2012)
miR-22	Decreased	P2X7	Excitability, inflammation, apoptosis	Leal et al. (2022)
miR-101a-3p	Decreased	c-Fos	Autophagy, apoptosis	Geng et al. (2021)
miR-135b-5p	Decreased	SIRT1	Apoptosis, proliferation	Li et al. (2020)
miR-15a-5p	Decreased	SIRT1, CXCL10	Apoptosis, inflammation	Li et al. (2020), Ma (2018)
miR-34a	Increased	Bcl-2, E2F1, MAP3K9	Apoptosis	Hu et al. (2012)
miR-183	Increased	Foxp1, p27	JAK/STAT signaling, neurogenesis	Wang and Zhao (2021), Feng et al. (2019)
miR-103a	Increased	BDNF	Neurogenesis, astrocyte activation, Inflammation	Zheng et al. (2019)
miR-134	Increased	Pum2, CREB, DCX	Neurogenesis, neural migration	Morris et al. (2019), Jimenez-Mateos et al. (2012)
miR-195	Decreased	NMDA-NR1, GluR2	Neurotransmission	Smirnova et al. (2005), Mao et al. (2020)
miR-219	Decreased	NR1	Neurotransmission	Hamamoto et al. (2020), Kocerha et al. (2009)
miR-128	Increased (acute seizure) Decreased(recurrent seizure)	SNAP-25, SYT1	Neurotransmission, synaptic formation	Wang et al. (2021)
miR-146a	Decreased	Notch-1	Inflammation, oxidative stress	Huang et al. (2019)
miR-142	Increased	PINK1	Mitochondrial autophagy, apoptosis	Ma (2018)

IL10, Interleukin 10; SPRY1, Sprouty RTK signaling antagonist 1; Fos, Fructo-oligosaccharides; SIRT1, silent mating type information regulation 2 homolog 1; CXC10, C-X-C motif chemokine ligand 10; BCL-2, B-cell lymphoma 2; PUM2, Pumilio2; DCX, doublecortin; SYT1, synaptotagmin-1; PINK1, PTEN-induced putative kinase 1.

During the progression of epilepsy, the miRNA changes in hippocampus may correlate with inflammatory responses. The dynamic linkage between interleukin-10 (IL-10) level,as a cytokine, and miR-187 level,as a post-transcriptional inflammation-related miRNA, has been evaluated in the hippocampus of a rat model of status epilepticus and patients with temporal lobe epilepsy. The hippocampal IL-10 protein level and miR-187 level were oppositely correlated following seizure epilepsy. In the hippocampus of patients, the expression of IL-10 was importantly increased, whereas miR-187 was decreased. Intrestingly, miR-187 level was decreased by IL-10 up regulation in an IL-10-dependent situation. The inhibition of miR-187 increased the expression of IL-10 in hippocampus of SE rats. These results suggest crucial involvement of miR-187 in the modulation of IL-10 anti-inflammatory effects on pathophysiological pathways of epilepsy (Alsharafi et al., 2015). mir-187 also inhibits IL-10 mediated inhibition of TNF-α, IL-6, and the p40 subunit of IL-12 (IL-12p40) which is secreted after stimulation of Toll-like receptor 4 (TLR4) (Rossato et al., 2012). Moreover, miR-27a-3p effectively promotes pathways to increase the expression levels of interleukin-1β (IL-1β), IL-6, and tumor necrosis factor-α (TNF-α) in the hippocampus of rat model of epilepsy (Lu et al., 2019). Additionally, microRNA-22 is protective against the development of epileptogenic connections in the brain via the inhibition of neuroinflammatory pathways. Moreover, loss of miR-22 increases the epileptic attacks (Almeida Silva et al., 2020).

Several miRNA changes in the hippocampus may associate with changes in apoptotic pathways in epilepsy. MiR-101a-3p decreased in hippocampus of epilepsy animal model. This miRNA protected neurons against apoptosis, reduced cell damages and autophagy via targeting c-FOS expression (Geng et al., 2021). Additionally, miR-135b-5p and miR-15a-5p were decreased in the hippocampus of epileptic rats as well as children with temporal lobe epylepsy. Both miR-135b-5p and miR-15a-5p modulate the apoptosis pathway via targeting SIRT1 expression. miR-135b-5p and miR-15a-5p can be promising indicator for diagnostic strategies in epileptic children (Li R. et al., 2020; Li N. et al., 2020). However, miR-34a is upregulated during seizure. This miRNA is involved in neuronal apoptosis and neurodegeneration through promoting signaling pathways related to caspase-3 protein. Targeting miR-34a microRNA resulted to the suppression of activated caspase-3, a protein is involved in enhanced neuronal survival and decreased neuronal apoptosis (Hu et al., 2012). The expression of miR-183 is also increased during pathological mechanisms of epilepsy. Suppression of miR-183 could induce the expression of FOXP1, that results to the suppression of the Jak/Stat signaling cascade and promotes neurogenesis, as well as suppression of neural damage in the hippocampus in epileptic rats, by which the epilepsy process could be inhibited (Feng et al., 2019). Increased level of miR-103a leads to apoptosis and BDNF depletion in the hippocampus tissues of epilepsy rats. Zheng research exhibited that supression of miR-103a can cease the inflammatory responses of astrocytes in the hippocampus and reduces the pathobiological apoptotic pathways in epileptic hippocampus by increasing the expression of BDNF (Zheng et al., 2019).

Higher levels of microRNA-134 have been found in various epileptic animal models and in desected brain tissue from temporal lobe epilepsy patients. Reduced microRNA-134 in the hippocampus can enhance seizure thresholds and ameliorate status epilepticus. Additionally, suppression of microRNA-134 following status epilepticus may significantly decreases the risk of recurrent seizures (Morris et al., 2019). Interestingly, miR-219 and miR-195 purportedly modulate the mediators of the excitatory neurotransmitter receptors NMDA-R1 and AMPA-GluR2 and the inhibitory neurotransmitter receptor GABA-A in the amygdala and the hippocampus of patients with mesial temporal lobe epilepsy. A reverse correlation between miR-219 and NMDA-NR1 levels is reported in both the amygdala and the hippocampus of epileptic cases in compared with controls. NR1 and GluR2 were over expressed, in line with low miR-195 levels in the neurons of epileptic patients (Hamamoto et al., 2020). The opposite correlation between miR-22 serum levels and ATP-gated ionotropic P2X7 receptors (P2X7R) levels in the hippocampus and neocortex of MTLE-HS cases has been reported, which proposes that evaluating serum miR-22 may be a potential indicator of P2X7R levels in brain of MTLE-HS cases. Crucially, decresed level of miR-22 in serum may be a precise biomarker of resistance to anti-epileptic drugs among MTLE-HS affected cases (Leal et al., 2022).

miR-132 expression increases in the pathobiology of epilepsy. This microRNA level changes in response to BDNF and fibrobast growth factor and regulates the expression of synaptic receptors including NR2A, NR2B, and GluR1 (Numakawa et al., 2011). Inhibition of miR-132 can protect hippocampal neurons against damages caused by epilepsy and elevated levels of miR-132 in neurons exacerbates neuronal damage following epilepsy (Qian et al., 2017). It has been shown that inhibition of miR-132 can decrease the mortality caused by pathological excitations of epileptic neurons (Peng et al., 2013). The expression of miR-132 increases in the latent phase of epilepsy, which can be investigated as a biomarker in the prediction and control of diseases. miR-132 also interact with other genes and factors in pathobiology of epilepsy (Qian et al., 2017). This microRNA binds to the 3’UTR binding sites of SOX11 transcription factor affecting neural differentiation and neuronal excitability in the setting of epilepsy (Haenisch et al., 2015). Overexpression of miR-132 increases voltage-gated calcium channels following epilepsy. Moreover, miR-132 induces epileptiform discharges and promotes epileptogenesis by reducing the activity of TrkB/BDNF pathway in the hippocampal neurons (Xiang et al., 2015). Interestingly, deletion of miR-132 led to a decrease in the number of neurons and mossy fiber sprouting. Silencing of miR-132 in astrocytes inhibited axonal growth and protects against neuronal damages induced by status epilepticus by inhibiting IL-1β effect on astrocytes polarization (Zhang et al., 2022).

## Hippocampal miRNAs in ischemic stroke

Ischemic stroke is a major public health concern and one of the most important causes of death in the world (Xu et al., 2018). Apoptosis process constitutes an important pathological part of brain damage following cerebral ischemia reperfusion injury (Campbell et al., 2019). Although, the accurate molecular mechanisms has yet to be fully characterize. A critical period of spontaneous recovery in the nervous system ensues immediately after the stroke, during which a maximal efforts occur to compensate the damages and to repair the neural injuries (Campbell et al., 2019). Following the stroke, there is a period of neurogenesis, angiogenesis, neural regeneration and synaptic plasticity in the central nervous system (Xu et al., 2018). Since neurogenesis in the brain is critical for recovery after the stroke, and the hippocampus is one of the niches of neurogenesis in the brain, many researches concentrated on neurogenesis in the hippocampus following stroke. In several studies, the role of microRNAs in recovery after stroke has been investigated.

Researches introduce neuroprotective effects of microRNAs against ischemia–reperfusion injuries. The level of miR-190 was dramatically reduced in the damaged area of brain in ischemia reperfusion (Xu et al., 2018; Jiang et al., 2021). Up regulation of miR-190 in the hippocampus enhanced neurogenesis through regulating Rho/Rho-kinase cascade. This miRNA improved neurological scores, brain edema, infarct volumes, and cell death following ischemia reperfusion (Jiang et al., 2021). The overexpression of miR-223 down regulates GluR2 and NR2B levels via binding to 3’-UTR of GluR2 and NR2B, suppresses NMDA-mediated calcium influx in the hippocampus, and preserved the hippocampal neurons against apoptosis caused by transient global ischemia (Harraz et al., 2012). Down regulation of miR-223 induces excitotoxic injury, which leads to increased expression of NR2B and GluR2, higher NMDA-mediated calcium influx, and enhanced small excitatory postsynaptic stimulations in the hippocampal neurons. Moreover, lack of miR-223 results in contextual memory impairments and increased neuronal apoptosis following transient ischemia and excitatory stimulations (Harraz et al., 2012). Data suggest that miR-23b is a key regulator of apoptosis via suppressing TAB3/NF-κB/p53 signaling cascade in hippocampus. The up regulation of miR-23b decreses TAB3 and NF-κB expressions in the hippocampus in ischemia reperfusion rats and preserved the neurons against neuronal death caused by acute ischemia reperfusion damage (Roshan-Milani et al., 2022). Additionally, miR-214 in the hippocampus is preservative against cerebral ischemia reperfusion damages and injection of miR-214 inhibitor in the hippocampus increased the volume of cerebral infarction and the neuronal loss in the hippocampus (Liu et al., 2021). This microRNA suppressed ROCK1 and protect neurons against apoptosis (Liu et al., 2021). The level of miR-210 was importantly increases following focal cerebral ischemia/reperfusion. Up regulation of miR-210 increased neovascularization and NPCs cumulating on the sidelines of ischemic lesion, proposing that miR-210 has a key role in neovascularization and NPC cumulating after focal cerebral ischemia/reperfusion (Meng et al., 2018). Moreover, miR-195 in the hippocampus has an important role in the microglial/macrophage polarization by regulating the connections between neurons and microglia via the modulation of CX3CL1 and CX3CR1. These results indicated that miR-195 could be a target to reduce neurodegeneration following chronic brain hypoperfusion (Mao et al., 2020).

Various studies introduce deleterious effects of microRNAs in ischemia–reperfusion. it has been found that miR-27b decreased AMPK expression and suppressed neurogenesis. In neural stem cells, inhibition of miR-27b increased the proliferation and differentiation of neurons through strengthening the AMPK pathway. In a mouse model of middle cerebral artery occlusion, administration of miR-27b inhibitor in the hippocampus stimulated neurogenesis and improved cognitive abilities after injury (Xu et al., 2018; Wang et al., 2019). Moreover, inhibition of miR-497 processing in animal model of ischemia increased neuronal viability by up regulation of anti-apoptotic protein, bcl-2 (Sinoy et al., 2017).

Madeline et al. showed that miR-9 expression is associated with neurogenesis and angiogenesis via the generation of neurons expressing vascular endothelial growth factor A (VEGF-A). They showed that miR-9 suppressed the transcription factors TLX and ONECUTs to modulate VEGF-A levels. Inhibition of miR-9 increases VEGF-A signaling in neurons, which causes thickening of the vessel wall to reach the physiological state of the neurovascular network in the central nervous system. Due to its bidirectional effects on neuronal proliferation and angiogenesis, miR-9 and its targets are potential candidates for the treatment of post-traumatic damage of the nervous system following stroke (Madelaine et al., 2017).

## Hippocampal miRNAs in pathobiology of schizophrenia

Schizophrenia is a debilitating disorder that has complex clinical manifestations including abnormal social behavior, mental disorder, cognitive deficits and emotional processing problems (Long et al., 2022). This psychological disorder affects approximately 1% of the world’s population. The exact cause of Schizophrenia is not fully understood. Improper synapses, especially changes in dopamine-glutamate transmission and abnormalities in the function of neural networks related to prefrontal cortex form the basis of the majority of schizophrenia symptoms (Heckers and Konradi, 2002). In recent years, connections between the hippocampus and the prefrontal cortex (PFC) have been identified given their roles in different cognitive and behavioral processes. Dysregulation in the hippocampal-prefrontal connections have also been showed in psychiatric disorders, most notably schizophrenia. The human hippocampus attains extremely processed inputs through the entorhinal and the prefrontal cortex and sends information back to the prefrontal, the entorhinal cortex and to the limbic system through direct projections (Sigurdsson and Duvarci, 2016). This unique network of interconnections effectively participates in at least two cognitive aspects; Memory and emotion processing, both of which are impaired in most schizophrenic patient (Heckers and Konradi, 2002).

Recent studies support the neurodevelopmental hypothesis in schizophrenia pathobiology, which proposes that genetic and environmental influences affect neurodevelopmental procedure that cause irregularity in both the brain synaptic plasticity and the neural network connectivity. These abnormalities result in schizophrenia in early adulthood. In this model, regulatory factors that govern specific pattern of gene expressions during neurodevelopment have important role in the pathological mechanism of schizophrenia (Heckers and Konradi, 2002; Miller et al., 2012; Loohuis et al., 2017; Gunasekaran et al., 2022). The effects of microRNA regulatory cascade in cognitive processing and brain network communication have attracted many researches. These potential effects are a milestone in sequential discovering of the molecular processes underlying development of several neuropsychiatric disorders, including schizophrenia.

MicroRNAs can regulate the expression of receptors and transcription factors and they are involved in signal transduction pathways in neurodevelopment of schizophrenia. microRNA-137 is abundant in the brain and has important effect on brain development and synaptic regulation (Loohuis et al., 2017). This microRNA acts as a key regulator in gene expression network, which is involved in the neurodevelopmental process rerated to schizophrenia (Loohuis et al., 2017). It has been reported that the miR-137 risk allele significantly associated with the early onset psychiatric complications in schizophrenia patients. Patients with schizophrenia who had the miR-137 risk genotype had smaller hippocampi and larger lateral ventricles (Lett et al., 2013; Patel et al., 2015). Studies on young people showed that miR-137 variant (rs1625579) affects brain connection networks between the hippocampus and the dorsolateral prefrontal cortex and disrupts cognitive abilities. Overexpression or inhibition of miR-137 causes disturbances in the function of neurons. Deletion of miR-137 in animal models causes defects in synaptic regulation, social connections, and cognitive behaviors (Cheng et al., 2018). Dysregulation of miR-137 in the brain cause psychiatric complications and emotional problems (Arakawa et al., 2019). Inhibition of miR-137 causes deficits in synaptic plasticity and abnormalities in dendrite development in the hippocampal neurons as well as behavioral problems including repetitive activities, cognition problems and Social behavior disorder (Cheng et al., 2018). Gene expression profile analyses showed that miR-137 targets phosphodiesterase 10a (PDE10A) expression (Cheng et al., 2018). Gene ontology analysis suggests that miR-137 regulates the expression of genes involved in neuronal differentiation, maturation processes and cell survival in the nervous system, all of which recognized to be vital for adequate neural network organization (Loohuis et al., 2017). It has been shown that miR-182 and miR-183 were both increased in the circulation of schizophrenia patients and hippocampus of schizophrenia rats. The miR-182/183 cluster could target DDC and suppress the expression of DDC. On the other hand, suppression of the miR-182/183 cluster improves schizophrenia symptoms and decreases apoptosis in the hippocamus. Additionally, it was reported that DCC expression is essential for the axon guiding pathway and synaptic formation in hippocampal neurons (Wang et al., 2022). Alteration in expression levels of other microRNAs including miR-296, miR-148b, miR-129-2, miR-223, miR-143, miR-144-3p, miR-132, miR-219 and miR-19-9 could cause abnormalities in neudevelopmental pathways in hippocampus in schizophrenia (Kocerha et al., 2009; Miller et al., 2012; Gunasekaran et al., 2022; Pan et al., 2023).

The level of miR-132 in the circulation of schizophrenic subjects is decreased compared to healthy subjects (Yu et al., 2015). Crucially, after the treatment with risperidone, the expression of miR-132 increases significantly, which indicates the importance of miR-132 as a biomarker in the diagnosis of schizophrenia. Imbalanced NMDA/AMPA receptor activity and expression play an important role in the pathophysiology of schizophrenia (Qian et al., 2017; Smigielski et al., 2020). miR-132 has been shown to induce schizophrenia-related synaptic plasticity through NMDAR expression. In addition, miR-132 contributes to impairments in neuronal differentiation and neurodevelopment in schizophrenia via several targets including DNMT3A, GATA2, and DPYSL3 (Balu et al., 2013; El Fatimy et al., 2018). It has been found that in the peripheral blood of schizophrenic patients, the expression level of BDNF is decreased, while miR-132 is up regulated. These findings show the involvement of miR-132-BDNF network in the pathophysiological mechanism of schizophrenia (Fu et al., 2022).

The expression level of miR-7 is higher in schizophrenia patients compared to the healthy group. This microRNA binds to SHANK3 mRNA and modulate the signaling pathways of the morphology and plasticity of neurons in the hippocampus. Moreover, the expression of three schizophrenia related genes (ERBB4, GABRA6, and GAD1) increases in correlation with the decreased levels of miRNA-7, and reduces in concert with the elevated levels of miR-7. The expression of another schizophrenia related gene,GRIN2A, elevated in concert with miR-7 up regulation and reduced when miRNA-7 was suppressed (Zhang et al., 2015). Taken together, this miRNA potentially is prominent in the pathophysiological pathways of schizophrenia (Horsham et al., 2015).

## Hippocampal miRNAs in other neurological disorders

In some disorders of the central nervous system, although the hippocampus does not play a central role, damage to the hippocampus along with damage to other brain areas can increase the complication. Alterations in the miRNA levels in the hippocampus can also be important in these diseases. For instance, during the pathological mechanism of Amyotrophic lateral sclerosis (ALS), the expression levels of miR-124a and miR-19b increased and the expression of miR-219 and miR-9 decreased. These changes in the microRNAs in the hippocampus are consistent with the increase in the number and differentiation of neural stem cells in the dendate gyrus region in the ALS brain (Marcuzzo et al., 2015; Martinez and Peplow, 2022). Moreover, miR-125b, has been shown to be significantly decreased in the SVZ and hippocampus in ALS. This microRNA was increased in motor neurons in ALS disease, and this increase is in line with decreased levels of Sox6 in the primary motor cortex. The overexpression of miR-125b in the primary motor cortex as well as the spinal cord is functionally associated with degeneration characteristic of the corticospinal tract and increased neurogenesis in the hippocampus (Marcuzzo et al., 2015).

Although the main area of brain damage in Parkinson’s disease (PD) is the basal ganglia, the hippocampus undergoes atrophy in PD. Khoo et al. showed that miR-1826 is elevated in the PD patients (Khoo et al., 2012). This miRNA is also increases in the circulating blood of patients affected with multiple sclerosis. This microRNA suppresses the expression of the neuronal PAS domain protein 3 gene and is involved in the regulation of neurogenesis, especially in the hippocampus (da Silva et al., 2016). Moreover, miR-193 dysrygulation is involved in development of PD through changes in PGC-1α/FNDC5/BDNF pathway (Baghi et al., 2021). This pathway has an important role in the functional regulation of the hippocampus (Liu et al., 2014).

Decreased hippocampal volume has been confirmed in migraine (Liu et al., 2018). The hippocampus is involved in pain perception and in pain-related attention and anxiety. Dysregulations in functional and structural connections between hippocampus and other brain regions has been reported in migraine (Planchuelo-Gomez et al., 2020; Zhu et al., 2021). The expression levels of miR-342-3p is elevated in migraine patients (Gallardo Lopez et al., 2023). miR-342-3p regulates proliferation and apoptosis pathways in hippocampus via targeting BCL-2 expression (Chen et al., 2021). Moreover, this miRNA increases the amyloid beta accumulation and contributes in AD pathology (Fu et al., 2019).

## Discussion and future perspective

miRNAs are involved in various features of hippocampal physiology, such as development, neurogenesis, and synapse formation. Moreover, dysregulation of miRNAs is contributed in the pathology of various hippocampus related disorders including epilepsy, Alzheimer’s disease and schizophrenia (Coolen and Bally-Cuif, 2015). Hence, the growing list of microRNAs as prominent biological regulators of neural processes is coming up. miR-138, miR-9-5p, miR-146a-5p, miR-124, miR-134, miR-137, and miR-184 are the most studied miRNAs in the hippocampus. Important pathways underlying neurodegenerative disorders, such as NF-Kβ, TNF-α pathways and Notch signaling cascade, are under the precise regulation of miRNAs (Schratt et al., 2006; Lang and Shi, 2012). In summary, it is essential to note that microRNAs do not have separated and isolated functions. The overlapping roles of miRNAs, suggested that they probably function in concert with each other to regulate neuronal activity.

There are strong documents that abnormalities in miRNAs are crucial in epileptic mechanisms through imbalanced activity of ion channels, inflammatory response, aberrant synaptic plasticity and neuronal apoptosis. Some miRNAs have been reported to affect molecular and cellular pathways involved in cognitive disorders including oxidative stress, inflammation, neural differentiation, migration and neurogenesis (Coolen and Bally-Cuif, 2015). Targeting these miRNAs is a considerable field for further approaches for regenerative therapies.

In the recent years, many studies have been reported the importance of miRNAs in the hippocampus. As post-transcriptional regulators of gene expression in different tissues at different stages, miRNAs definitely are key elements to govern the hippocampal development and disorders. However, research to determine the effect of microRNAs on developing and damaged hippocampal tissue is still in its nascent stages. Considering that, specific miRNAs participate in different regulatory networks during development and have synergistic or antagonistic functions; their precise molecular mechanism in the context of complex communications in brain structures is very intricate and multifaceted. This is especially the case when considering the specific miRNAs as biomarkers related to pathological conditions of nervous system. Therefore, accurate interpretation of miRNA expression and function, is very important not only for investigating their position as biomarkers in various physical and mental conditions, but also for considering as an important candidate in therapeutic strategies (Cho et al., 2019).

Given that miRNAs can target multiple mRNAs, their manipulation provides the innovated, multi-targeted attitude to modify gene expression networks in disease. Targeting different miRNAs has also been applied to fine-tune specific molecular pathway, enabling targeted therapeutic approaches for nervous system disorders. An interesting strategy to ensure targeted delivery is the rapidly advancing field of investigations relating exosome-based miRNA treatment against neurodegenerative disorders (Cho et al., 2019; Sudhakar and Richardson, 2019). Exosomes are the key carriers of circulating miRNAs. They naturally released to exchange miRNAs between cells. Exosomes easily cross the blood–brain barrier and integrate with the membrane of target cells (Cho et al., 2019). Research on exosome-based miRNA is promising not just as the content of microRNA biomarkers, but also as a strategy to deliver miRNA-based treatments to the nervous system. Understandably, the future advances in this field have the promising potency to pave the way for emerging treatments of neurological disorders.

## Author contributions

SR contributes in literature collection and manuscript writing. AK reviewed and corrected the manuscript grammatically. EB and SR participated in the literature collection. MD conducted the literature designing, writing, editing, and final reviewing of the manuscript. All authors contributed to the article and approved the submitted version.

## Conflict of interest

The authors declare that the research was conducted in the absence of any commercial or financial relationships that could be construed as a potential conflict of interest.

## Publisher’s note

All claims expressed in this article are solely those of the authors and do not necessarily represent those of their affiliated organizations, or those of the publisher, the editors and the reviewers. Any product that may be evaluated in this article, or claim that may be made by its manufacturer, is not guaranteed or endorsed by the publisher.
